# Impacts of the COVID-19 pandemic on life expectancy at birth in Asia

**DOI:** 10.1186/s12889-023-16426-9

**Published:** 2023-08-09

**Authors:** Yan Mo, Qiushi Feng, Danan Gu

**Affiliations:** 1 Centre for Family and Population Research, National University of Singapore, Singapore, Singapore; 2 Department of Sociology and Anthropology, Centre for Family and Population Research, National University of Singapore, Singapore, Singapore; 3 Population Division, DESA, United Nations, New York, USA

**Keywords:** COVID-19 pandemic, Asia, Life expectancy at birth, Decomposing life expectancy, Excess deaths

## Abstract

**Objective:**

To investigate the impact of the COVID-19 pandemic on life expectancy at birth (*e*_*0*_) for 51 Asian countries and territories from January 1, 2020 to December 31, 2021.

**Method:**

Based on age-sex-specific mortality used for estimating the changes in *e*_*0*_ for years 2019, 2020, and 2021 from the 2022 revision of the World Population Prospects, we employed Arriaga’s discrete method to decompose changes in *e*_*0*_ into both absolute and relative contributions of changes in age-specific death rate, and further obtained the age-sex-specific contribution to changes in *e*_*0*_ by country/territory and period (i.e., 2019–2020 and 2020–2021) for Asia.

**Findings:**

The COVID-19 pandemic reduced 1.66 years in *e*_*0*_ of the Asian population from 2019 to 2021, slightly lower than the world average of 1.74 years. South Asia had a high loss of 3.01 years, whereas Eastern Asia had almost no changes. Oman, Lebanon, India, Armenia, Azerbaijan, Indonesia, and the Philippines experienced a high loss of above 2.5 years in *e*_*0*_. Despite significant national and territorial variations, the decline of* e*_*0*_ in Asia was mostly from the age group of 60–79 years, followed by age groups of 80 + and 45–59 years; and age groups of children contributed little (i.e., 0–4 and 5–14 years old). Males suffered more losses than females in this pandemic. Asian nations saw less loss in *e*_*0*_ in the second year of the pandemic, i.e., 2020–2021, than in the first year, i.e., 2019–2020, but this recovery trend was not observed in Southern Asia and South-Eastern Asia. Countries from Central Asia and Western Asia, such as Kazakhstan, Armenia, Azerbaijan, Lebanon, and Oman, had extraordinarily more losses in *e*_*0*_ in the first year at ages around 70.

**Conclusion:**

The COVID-19 pandemic had significantly affected *e*_*0*_ of Asian populations, and most contribution to the reduction of *e*_*0*_ came from the three older age groups, 60–79 years, 80 + years, and 45–59 years, with great variations across countries/territories. Our findings could have important implications for development of more resilient public health systems in Asian societies with better policy interventions for vulnerable demographic groups.

**Supplementary Information:**

The online version contains supplementary material available at 10.1186/s12889-023-16426-9.

## Introduction

The COVID-19 pandemic has lasted more than three years, with the official death toll of 6.84 million as of 16 February of 2023 [1]. This number, however, likely underestimates the true impact of COVID-19 on mortality, due to challenges in accurately identifying COVID-19-relateds deaths, incomplete registration systems in many countries and areas, and the undercounting of deaths indirectly caused by the pandemic. The inconsistent definitions of COVID-19 deaths across countries further prevent the use of official statistics for international comparisons [2, 3].

The use of excess deaths could address these limitations, which reveals the number of deaths exceeding the expected level projected in the absence of this pandemic [3]. According to the World Health Organization (WHO) and the United Nations Department of Economic and Social Affairs (UN DESA) joint Technical Advisory Group for COVID Mortality Assessment (thereafter WHO-UN COVID TAG) [3, 4], excess deaths not only encompass fatalities directly attributed to the COVID-19 virus but also account for indirect deaths resulting from factors such as limited access to medical care during outbreaks, as well as the reduced mortality due to fewer cases of seasonal influenza, traffic accidents, or occupational injuries during the pandemic [3]. The WHO-UN COVID TAG reported the number of global excess deaths due to the COVID pandemic was 14.9 million in 2020 and 2021, nearly three times the official death toll [3].

The COVID-19-related mortality varies by age and sex [3, 5–9], and such associations also change by the pandemic stage [10–13]. The sheer number of excess deaths is thus affected by the age and sex structures of a given population, making a direct comparison between nations and territories less meaningful. Calculating changes in sex-specific life expectancy during the pandemic may be more appropriate as it is independent of population age and sex structures. This method is suitable for international comparisons and provides insights into the impact of COVID-19 on mortality [10].

A large number of studies have estimated changes of life expectancy in this pandemic [14–21]. By incorporating the WHO-UN TAG’s age- and sex-specific estimates for excess deaths for each country, the 2022 revision of the World Population Prospects (thereafter WPP 2022) by the UN DESA, Population Division, reveals a global decrease of 1.74 years in life expectancy at birth (*e*_*0*_) for both sexes from 2019 to 2021, with 1.80 years for males and 1.61 years for females, respectively [9]. Such declines were primarily attributable to the increased mortality among individuals aged 60 or older with 65% for males and 72% for females [10].

To our knowledge, only two studies have thus far examined changes in life expectancy during the pandemic and analyzed the variations by age and sex for Asia and its countries and territories [10, 22]. However, one focused solely on the changes in life expectancy at age 60 [10], whereas the other only investigated the changes in *e*_*0*_ for India in 2020 [22]. An examination of the age-sex-specific contribution to changes of *e*_*0*_ for Asian countries and territories could help fill the gap and shed light on understanding how the pandemic affected human mortality differently in Asian populations and help identify vulnerable groups for policy interventions. Moreover, as Asian countries and territories differed significantly during the pandemic in vaccination and containment policies [3, 4, 23–25] with varying infection rates and vaccination rates at different stages of the pandemic [11, 26–29], further analyses by region and period are necessary. Based on the WPP 2022 estimates, this study decomposed the changes in *e*_*0*_ from 2019 to 2021 to show the age-sex-specific contribution by region, country and territory, and period (i.e., the years 2020 and 2021) for Asia.

## Methods

The age-sex-specific mortality that were used for estimating the changes in* e*_*0*_ in 51 Asian countries and territories for years 2019, 2020, and 2021 were obtained from WPP 2022 [9]. WPP 2022 incorporated the WHO-UN COVID TAG estimates on excess deaths from the COVID-19 pandemic for all countries of the world in 2020 and 2021 [4, 9, 18]. The detailed methodologies used by the WHO-UN COVID TAG and the WPP 2022 estimates are available on the WHO website [4] and the UNDESA Population Division website [9, 30, 31]. With these annual age-sex-specific mortality schemes, we employed the widely used Arriaga’s discrete method [20, 32] to decompose the changes in *e*_*0*_ into both absolute and relative contributions of changes in age-specific death rates between two time points [33]. The formula is as follows:

1 $${e}_{0}^{t2}-{e}_{0}^{t1}= {\sum }_{x=0}^{\omega -1}[\frac{{l}_{x}^{t1}}{{l}_{0}^{t1}}(\frac{{L}_{x}^{t2}}{{l}_{x}^{t2}}-\frac{{L}_{x}^{t1}}{{l}_{x}^{t1}})+ \frac{{T}_{x+1}^{t2}}{{l}_{0}^{t1}}(\frac{{l}_{x}^{t1}}{{l}_{x}^{t2}}- \frac{{l}_{x+1}^{t1}}{{l}_{x+1}^{t2}})]$$

where* l*_*0*_ denotes the life table radix, and* l*_*x*_ refers to the number of survivors at the exact age *x* given the radix *l*_*0*_; *L*_*x*_ denotes the number of person-year lived at age* x*; *T*_*x*_ refers to the number of cumulative person-year lived at age *x* and beyond; *e*_*0*_ denotes the number of years of life expectancy at birth; and ($\omega -1)$ presents the last age group (i.e., 100 + years in this study). The first component in the right side of the equation $\frac{{l}_{x}^{t1}}{{l}_{0}^{t1}}\left(\frac{{L}_{x}^{t2}}{{l}_{x}^{t2}}-\frac{{L}_{x}^{t1}}{{l}_{x}^{t1}}\right)$ refers to the number of years of the direct contribution of mortality change at age *x* to the changes in *e*_*0*_, whereas the second part $\frac{{T}_{x+1}^{t2}}{{l}_{0}^{t1}}(\frac{{l}_{x}^{t1}}{{l}_{x}^{t2}}- \frac{{l}_{x+1}^{t1}}{{l}_{x+1}^{t2}})$ represents the number of years of the indirect contribution of mortality change at age *x* to the changes in *e*_*0*_. For the last age group ($\omega -1)$, there is no second component. After calculating the contribution of each age from Eq. (1), we further calculated the cumulative contribution to the changes in *e*_*0*_ for any age range and the relative contributions of each age (or any age range).

In the present study, we presented both the annual and the biennial sex-specific absolute and relative contributions by mortality changes for age groups of 0–4, 5–14, 15–44, 45–59, 60–79, and 80 + years to changes in *e*_*0*_ from January 1, 2020 to December 31, 2021.

## Results

**Fig. 1:**
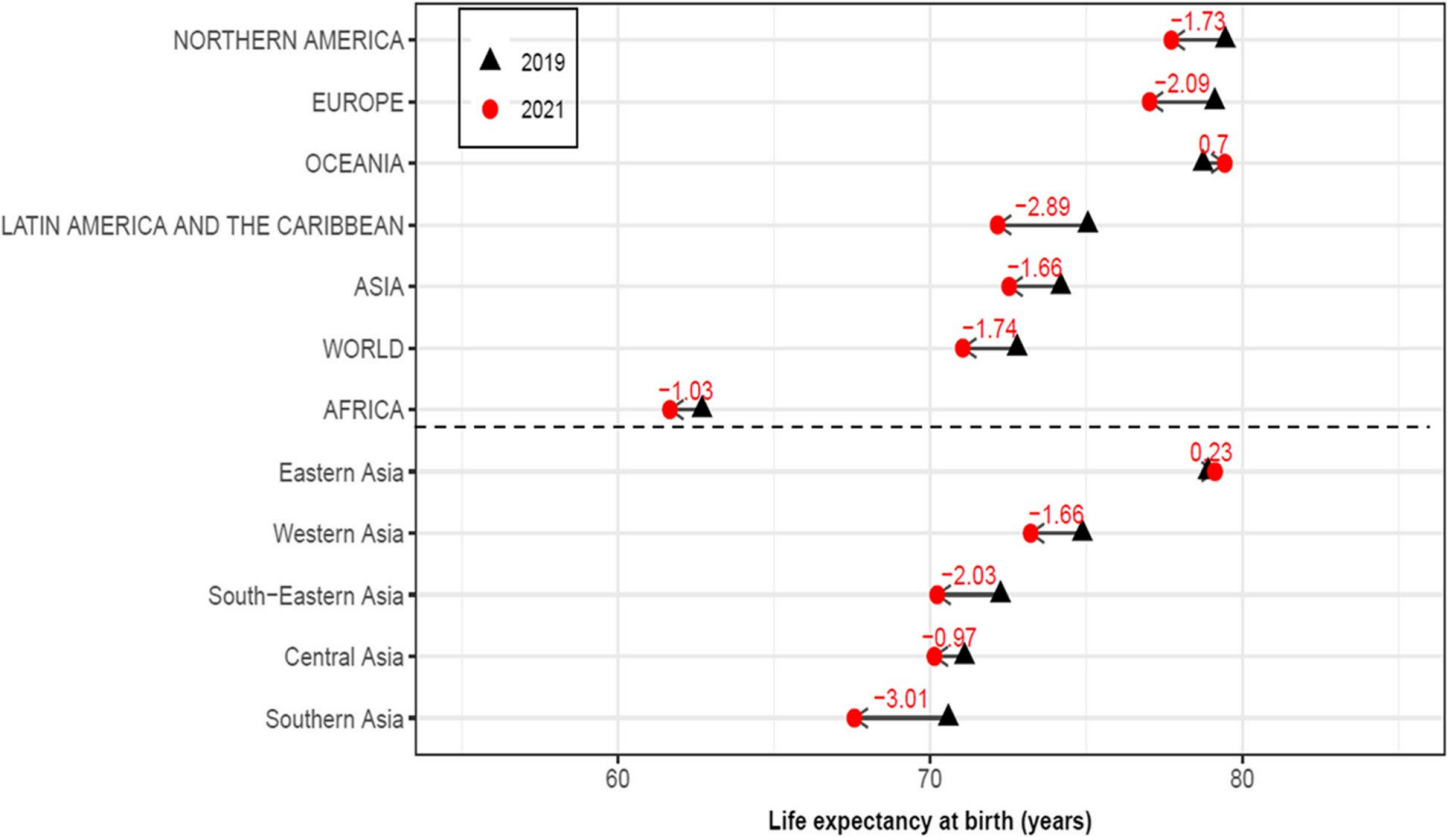
Life expectancy at birth for the world, regions, and the subregions of Asia, both sexes combined, 2019 and 2021

Figure 1 shows that *e*_*0*_ for both sexes combined had a decline in 2021 for the world and in most regions compared to 2019, with a reduction of 1.74 years for the world and a reduction of 1.66 years for Asia. Within Asia, Southern Asia and South-Eastern Asia declined by 3.01 and 2.03 years, respectively; by contrast, *e*_*0*_ in Eastern Asia increased slightly.

Table 1 displays age-sex-specific contributions to the changes in *e*_*0*_ from 2019 to 2021 by region. The global *e*_*0*_ experienced a decline by 1.6 and 1.8 years for females and males, respectively, most of which came from the age groups of 60–79 (0.8 years for females and 0.9 years for males) and 45–59 (0.4 years for females and 0.5 years for males). This pattern is generally applicable to Asia. With an exception for Eastern Asia, the age pattern of contribution to changes in *e*_*0*_ was similar across Asian subregions, although their magnitudes of contribution varied by different age groups.

**Table 1:** Contribution of age-specific mortality to changes in life expectancy at birth (*e*_*0*_) by sex and region, 2019–2021

Region or subregion	*e*_*0*_ in 2019 (Years)	Changes in *e*_*0*_ in 2019–2021 (Years)	Age groups (Years)					
			0–4	5–14	15–44	45–59	60–79	80 +
**Females**								
**World**	**75.4**	**-1.6**	**0.1**	**0.0**	-**0.2**	**-0.4**	**-0.8**	**-0.3**
Africa	64.6	-0.9	0.3	0.1	0.0	-0.3	-0.7	-0.2
**Asia**	**76.7**	**-1.6**	**0.1**	**0.0**	**-0.2**	**-0.3**	**-0.9**	**-0.3**
Europe	82.3	-1.8	0.0	0.0	-0.3	-0.3	-0.7	-0.5
Latin Am. & Caribbean	78.3	-2.5	0.1	0.0	-0.4	-0.7	-1.2	-0.3
Northern America	82.0	-1.3	0.0	0.0	-0.2	-0.4	-0.6	-0.1
Oceania	81.3	+ 0.3	0.1	0.0	0.3	0.1	0.0	-0.1
Eastern Asia	81.9	+ 0.3	0.1	0.0	0.0	0.1	0.1	0.0
South-Eastern Asia	75.1	-2.0	0.1	0.0	-0.2	-0.4	-1.0	-0.5
Southern Asia	72.4	-2.9	0.2	0.0	-0.3	-0.7	-1.5	-0.6
Central Asia	74.2	-1.0	0.1	0.0	0.0	-0.2	-0.8	-0.1
Western Asia	77.7	-1.6	0.0	0.0	-0.0	-0.3	-0.9	-0.4
**Males**								
**World**	**70.2**	**-1.8**	**0.1**	**0.0**	**-0.3**	**-0.5**	**-0.9**	**-0.3**
Africa	60.8	-1.1	0.3	0.1	-0.1	-0.4	-0.8	-0.2
**Asia**	**71.8**	**-1.7**	**0.1**	**0.0**	**-0.2**	**-0.4**	**-0.9**	**-0.3**
Europe	75.8	-2.2	0.0	0.0	-0.5	-0.5	-0.9	-0.3
Latin Am. & Caribbean	78.3	-2.5	0.1	0.0	-0.7	-0.9	-1.3	-0.3
Northern America	77.0	-2.1	0.0	0.0	-0.5	-0.5	-0.8	-0.2
Oceania	76.3	+ 1.0	0.1	0.0	0.6	0.3	0.1	0.0
Eastern Asia	76.1	+ 0.2	0.1	0.0	0.1	0.0	0.0	0.0
South-Eastern Asia	69.5	-2.0	0.1	0.0	-0.2	-0.5	-1.1	-0.3
Southern Asia	68.9	-3.1	0.2	0.0	-0.4	-0.8	-1.5	-0.5
Central Asia	68.0	-0.9	0.1	0.0	0.1	-0.2	-0.8	-0.1
Western Asia	72.2	-1.6	0.2	0.0	0.1	-0.4	-1.1	-0.3

*Note*: A positive sign implies a gain in life expectancy or an improvement in mortality. Only countries or areas with 100,000 inhabitants on January 1, 2022, were included. The sum of all age groups may not be equal to the total due to rounding

Source: Authors’ calculation based on life tables from the World Population Prospects 2022 [30]

**Fig. 2:**
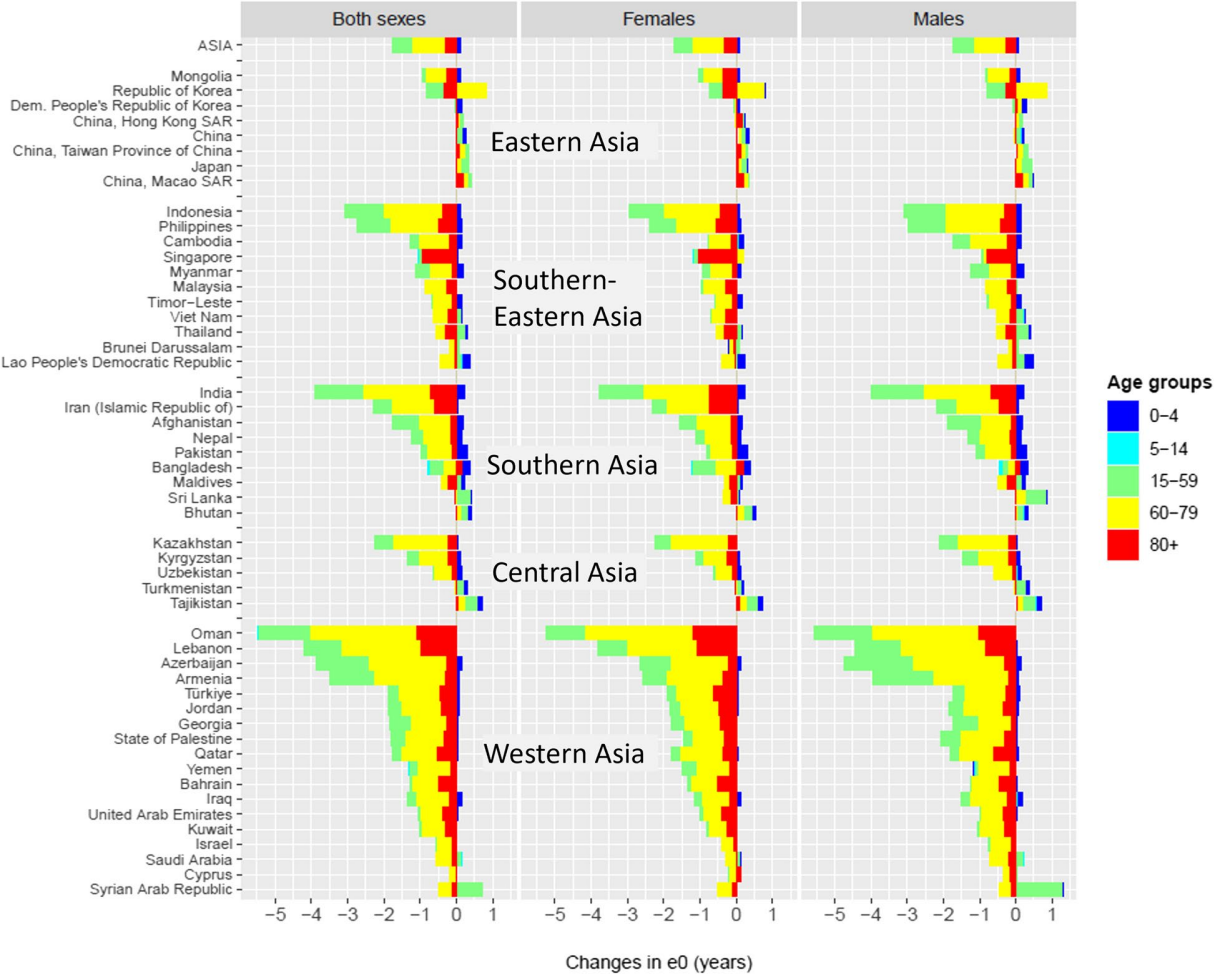
Contributed years to changes in life expectancy at birth (*e*_*0*_) by age group and sex for Asian country/territory classified by subregion, 2019–2021

We then calculated the age-specific contributions to the changes in *e*_*0*_ for each of all 51 Asian countries and territories from 2019 to 2021 (Fig. 2). For the six age groups analyzed (i.e., 0–4, 5–14, 15–44, 45–59, 60–79, and 80 + years), the most contribution to the reduction in *e*_*0*_ was primarily from ages 60–79 years, followed by the age groups 80 + and 45–59 years. The first two age groups (i.e., 0–4 and 5–14 years) made a very limited contribution to the reduction in *e*_*0*_, and in some cases, the mortality rate in age group 0–4 years witnessed even an improvement, which positively contributed to *e*_*0*_.

A significant variation in the age pattern of contribution to changes in *e*_*0*_ was observed, however. Among Asia’s 51 countries and territories, Oman, Lebanon, India, Armenia, Azerbaijan, Indonesia, and the Philippines stood out with the largest decrease in *e*_*0*_ ,with above 2.5 years from 2019 to 2021, whereas almost all Eastern Asian countries and territories saw a relatively minor change in *e*_*0*_. For most countries in Asia, high mortality rates among older adults in the age group of 60–79 years usually contributed the most to the reduction in *e*_*0*_; however, Singapore, Thailand, and the Republic of Korea were exceptional, where the age group of 80 + years played a major role. It is also noteworthy that, among the countries with the largest decline in *e*_*0*_, the age group of 45–59 years made a sizeable contribution, especially in males.

Furthermore, within Asian subregions, the age-specific contribution to the changes in *e*_*0*_ also varied considerably. Taking South-Eastern Asia countries (or the Association of South-East Asian Nations, ASEAN) as an example, Indonesia and the Philippines had the largest reduction in* e*_*0*_ with 2.5–3.0 years losses for both sexes combined. The attributable reduction in *e*_*0*_ by 45–59 years in these two ASEAN countries was sizeable, around 20–30% for males, females, and two sexes combined. By contrast, the reduction in *e*_*0*_ in Singapore was primarily from increased mortality at ages 80  years and beyond; and the total reduction in *e*_*0*_ in Brunei Darussalam and the Lao People’s Democratic Republic was very minor. Another example is in Western Asia: the male mortality for ages 15–44 years in Syrian Arab Republic decreased due to the temporary ceasefire, contributed 1.2 years  to *e*_*0*_ in 2021 compared to 2019, while mortality rate in the same age group in Oman and Lebanon witnessed a substantial increase, contributed 0.6 and 0.4 years to their respective *e*_*0*_ in 2019-2021.

Table 2 compares the changes in *e*_*0*_ in the two periods: 2019–2020 and 2020–2021 for 51 Asian countries and remaining 149 countries of the world. In the first year of the pandemic (i.e., 2019–2020), about 20% of the 51 Asian countries and territories experienced a reduction of more than 1.5 years in *e*_*0*_ for both males and females, compared to the previous year; in the second year of the pandemic (i.e., 2020–2021), this figure fell considerably to approximately 10%. Meanwhile, the proportion of countries with * e*_*0*_ increasing more than 0.3 years in the second year almost doubled, rising from approximately 10% (5 countries) to 20% (10 countries). A further check shows that such *e*_*0*_ increases in the second year among these 10 Asian countries were mainly driven by the reduced reduction in mortality in the age group of 60–79 years, which accounted for nearly half of the increase in *e*_*0*_. The patterns presented above suggest that the excess mortality observed in Asian countries and territories during the first year was reduced in the second year.

**Table 2:** Percentage distribution of countries with different magnitudes in changes in life expectancy at birth in 2020 and in 2021 compared to their respective previous year

Changes in *e*_*0*_	% Countries (2020 compared to 2019)			% Countries (2021 compared to 2020)		
	Both sexes	Females	Males	Both sexes	Females	Males
**Among 51 Asian countries**						
≤ -1.5 years	21.6	19.6	23.5	9.8	9.8	9.8
-1.5 – -0.5 years	25.5	29.4	27.5	29.4	29.4	25.5
-0.5 – 0 years	19.6	13.7	15.7	19.6	21.6	19.6
0 – 0.3 years	23.5	27.5	23.5	23.5	19.6	25.5
≥ 0.3 years	9.8	9.8	9.8	17.6	19.6	19.6
**Among the remaining 149 countries**^**a**^						
≤ -1.5 years	10.1	9.4	13.4	19.5	20.1	18.8
-1.5 – -0.5 years	39.6	33.6	40.3	30.9	32.9	30.9
-0.5 – 0 years	28.2	28.9	26.2	23.5	20.8	26.9
0 – 0.3 years	14.8	21.5	11.4	16.1	12.1	13.4
≥ 0.3 years	7.4	6.7	8.7	10.1	14.1	10.1

Source: Calculated from the World Population Prospects 2022 [30]

^a^Only countries or areas with 100,000 inhabitants on January 1, 2022, were included

Table 2 also shows the rest part of the  world, which did not see the exactly same pattern as Asia. Among the 149 non-Asian countries and territories of the world, about 10% underwent declines in *e*_*0*_ by more than 1.5 years in the first year, and this number almost doubled in the second year. But similar to Asia, as compared to the first year of the pandemic, the number of countries and territories with growing *e*_*0*_ increased in the second year.

**Fig. 3:**
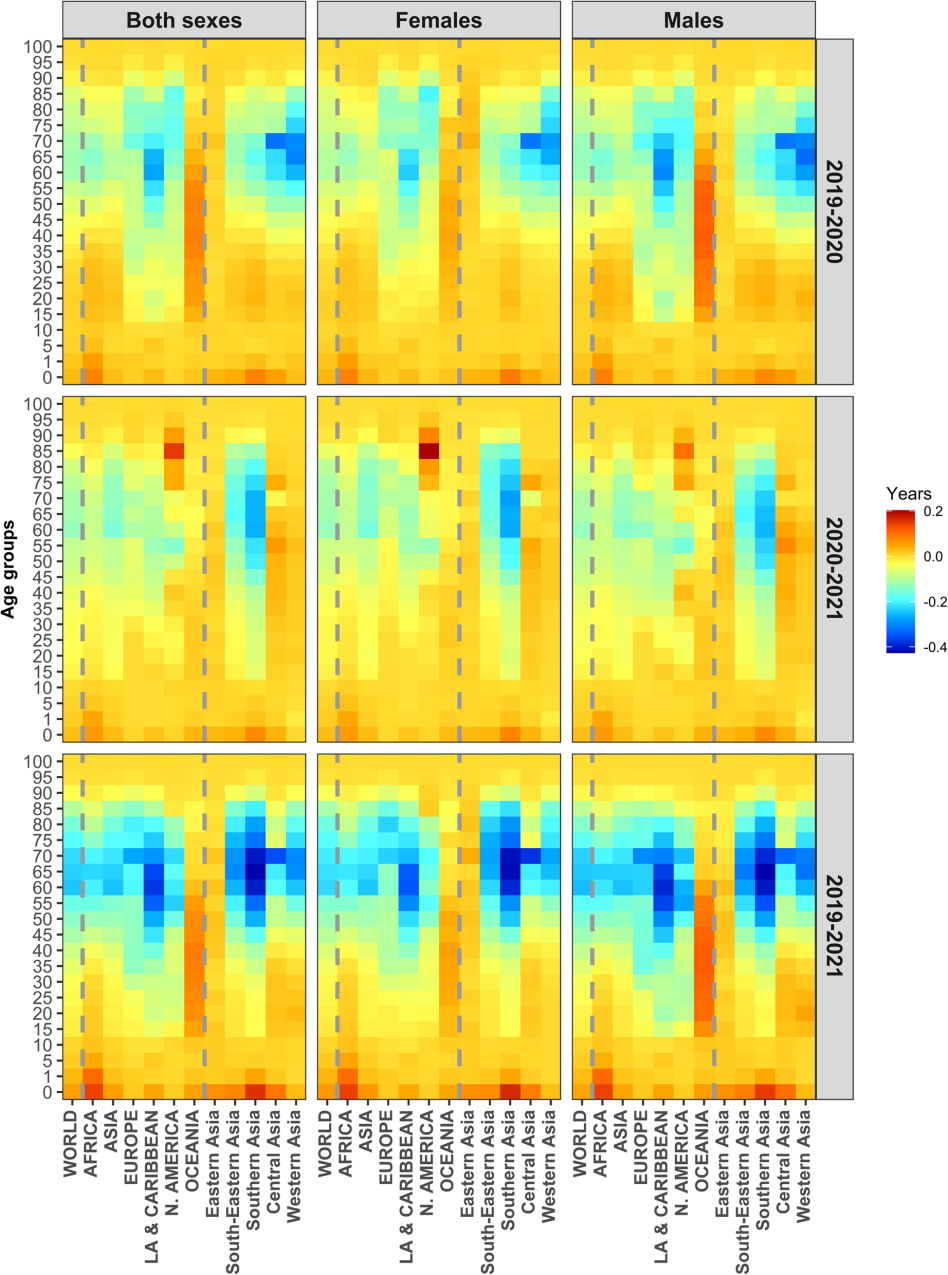
Contributed years to changes in life expectancy at birth (*e*_*0*_) by age group, sex, region, and period (2019–2020, 2020–2021, and 2019–2021)

Figure 3 further presents contributed years to the changes in *e*_*0*_ in 2019–2020, 2020–2021, and 2019–2021 for the world, regions, and Asian subregions by sex and age group, respectively. The graph clearly reveals that the changes in* e*_*0*_ varied greatly across different age groups, regions of the world, and subregions of Asia, and between the two periods ( 2019–2020 vs 2020–2021). Across the world, the first year of the pandemic usually witnessed more losses in* e*_*0*_ than the second year; however, an opposite pattern was observed for Southern Asia and South-Eastern Asia, both of which experienced more losses in the second year. In addition, Central Asia and Western Asia experienced significantly big losses in the first year at ages around 70 years for both females and males. Figure 3 further shows that the reduction in *e*_*0*_ in the second year was still mainly driven by the age groups from 60–64 to 75–79 years. These age groups accounted for nearly half of the reduction, although such shares reached 70–80% in the first year. With few exceptions, males and females shared similar age-specific reduction patterns within each region or subregion. A more or less similar pattern was also found in most Asian countries or territories (see Figure A1 in Appendix).

Table 3 shows the male-to-female sex ratio of the reductions in *e*_*0*_ from 2019 to 2021 and the sex ratio of these reductions to *e*_*0*_ in 2019. The sex ratios for the world and all continents were greater than 1, suggesting that males experienced greater losses in *e*_*0*_ than females in both absolute and relative terms. Among Asian subregions, except for Eastern Asia and Central Asia, all other subregions had a sex ratio greater than 1 as well.

**Table 3:** The male-to-female sex ratio of the reduction in life expectancy at birth (*e*_*0*_), 2019–2021

Region or subregion	*Sex ratio in reduced years in e*_*0*_,*2019–2021*	*Sex ratio in reduced years in e*_*0*_* relative to its base in 2019*
**World**	**1.12**	**1.20**
Africa	1.23	1.31
**Asia**	**1.24**	**1.11**
Europe	1.26	1.35
Latin America and the Caribbean	1.56	1.37
Northern America	1.03	1.66
Oceania	3.30	3.52
Eastern Asia	0.57	0.61
South-Eastern Asia	1.01	1.09
Southern Asia	1.05	1.10
Central Asia	0.93	1.01
Western Asia	1.01	1.08

Source: Calculated from the World Population Prospects 2022 [30]

## Discussion

The COVID-19 pandemic has imposed disastrous impacts on life and health of human beings worldwide. The global reduction in life expectancy in this pandemic may cause the first interruption of the increasing trend of global life expectancy since the year of 1950 [18]. As the world is currently seeing a gradual transition to the post-pandemic era, now comes a good timing to evaluate the impact of this pandemic on population health. By using the latest revision of WPP 2022 released by the United Nations, and focusing on Asia, the most populous continent in the world, this paper analyzed how different sexes and age groups contributed to changes in *e*_*0*_ during the pandemic, at different periods, for its subregions, and for 51 Asian countries and territories.

As revealed by this study, although Asia as a whole had a relatively smaller loss in *e*_*0*_ from 2019 to 2021 compared to other continents, Southern Asia suffered one of the largest losses in *e*_*0*_ in the world. Meanwhile, there was a great variation within Asia; in contrast to Southern Asia, Eastern Asia appeared to be one of the least affected regions in the world for the first two years of the pandemic. To elucidate the impact of COVID-19 on Asian populations, this study conducted the age-sex-specific decomposition of the changes in *e*_*0*_. Similar to other parts of the world, we found that the greatest reduction in *e*_*0*_ among Asian populations was primarily attributable to the mortality increase in the age group of 60–79 years, followed by two age groups of 80 + and 44–59 years.

The disproportionate impact that the COVID-19 pandemic has had on various communities underscores the need for a well-functioning public health system. As COVID-19 virus proves to be more threatening toward people at older ages and males, who are biologically frailer in the immune system [34], many deaths in the young older age group (65-79 years) and the adulthood group (15-59 years) should be preventable given a more robust and effective public health system. As shown in our analysis, the *e*_*0*_ reduction in Singapore, renowned for its advanced healthcare system [35], concentrated on the oldest-old populations. As suggested in literature, to response to this pandemic depends on the promptness of public interventions like lockdown enforcement and mass vaccination [36–39]. The pandemic also brought to the fore the strain on healthcare resources when medical services were overwhelmingly directed toward COVID-19 patients [40], and older adults may suffer from chronic conditions such as diabetes, heart disease, and chronic respiratory illnesses in many countries [41–43]. An effective healthcare system should respond relatively well to these challenges or crises, as shown by the good performance of Eastern Asian countries in our analysis. In this sense, our results align with previous studies highlighting the importance of healthcare accessibility and resource availability within the public health system in mitigating reduction of life expectancy during a pandemic [44, 45].

The effort of this study to reveal how the COVID pandemic impact was distributed among different age and sex groups provides useful clues to identify vulnerable demographic groups for policy interventions. Yet, further investigations are also needed to expand to include more factors. Plenty of evidence from different populations and geographic regions suggested that COVID-19 infections and mortality risk were associated with both macro-level factors (such as development levels, poverty, capacity of public healthcare system, healthcare equity, environmental characteristics, population density and composition, policy responses, vaccination coverage, culture) and individual factors (such as age, sex, socioeconomic status, race/ethnicity, lifestyles, health literacy, disease conditions) [5, 34, 46–53]. Understanding more of these affecting factors would help to prevent a natural disaster from turning into a social disaster, a key lesson the world could learn from this COVID pandemic [54]. In addition, the future analyses of the COVID-19 mortality impact, if considering a country or territory’ specific stage of epidemiological transition or demographic transition, could obtain extra theoretical and practical significance. Lastly, plenty of literature on the COVID impact on life expectancy did not pay much attention to the period difference. By comparing the two periods, 2019–2020 and 2020–2021, this study presented interesting temporal patterns of age-sex-specific contributions to the changes in *e*_*0*_. We highly recommend future studies incorporate this important dimension in analysis.

This study has a few limitations. The data on the impacts of this pandemic on human life expectancy remains incomplete by far, owing to gaps and time lags in data collection and dissemination. Despite these challenges, WPP 2022 still accounted for the impact of the COVID-19 pandemic on population and demographic estimates with the latest available evidence for the years 2020 and 2021. There is an ongoing debate on the methodology and the outcome of excess deaths in this field [55], and the WPP estimates of excess deaths due to the COVID-19 pandemic still needs to be further improved with more data sources available in its 2024 Revision. As a result, the full impact of the pandemic on demographic and socioeconomic domains may not be known unless the data limitations and methodological debate are solved. Second, this study did not cover the most recent development of the COVID pandemic in Asia for the year 2022. The significant rise of COVID cases in Eastern Asian nations is not discussed, especially the recent relaxation of China’s COVID policy since the late 2022 [56, 57]. A complete report on the impact of the COVID-19 pandemic on life expectancy in Asia is expected to be done in the near future.

### Supplementary Information

**Additional file 1: Appendix Figure A1. **Contributed years to the changes in life expectancy at birth (*e*_*0*_) by age group and Asian country/territory, both sexes combined, 2019-2021

## Data Availability

The life tables used in this data are from the World Population Prospects 2022. United Nations, Department of Economic and Social Affairs, Population Division, New York. They can be downloaded at https://population.un.org/wpp/.
