# Supplementary material for: Impacts of the COVID-19 pandemic on life expectancy at birth in Asia
Source: BMC Public Health. 2023 Aug 9;23:1508. doi: 10.1186/s12889-023-16426-9 (PMC10410782; doi:10.1186/s12889-023-16426-9)
Supplement: Supplementary file 1 — Additional file 1: Appendix Figure A1. Contributed years to the changes in life expectancy at birth (e0) by age group and Asian country/territory, both sexes combined, 2019-2021 [file 12889_2023_16426_MOESM1_ESM.docx]

**Appendix**

**Figure A1.** Contributed years to the changes in life expectancy at birth (*e_0_*) by age group and Asian country/territory, both sexes, 2019-2021

Southern

Asia

Central

Asia

South-Eastern Asia

Western Asia

Eastern Asia


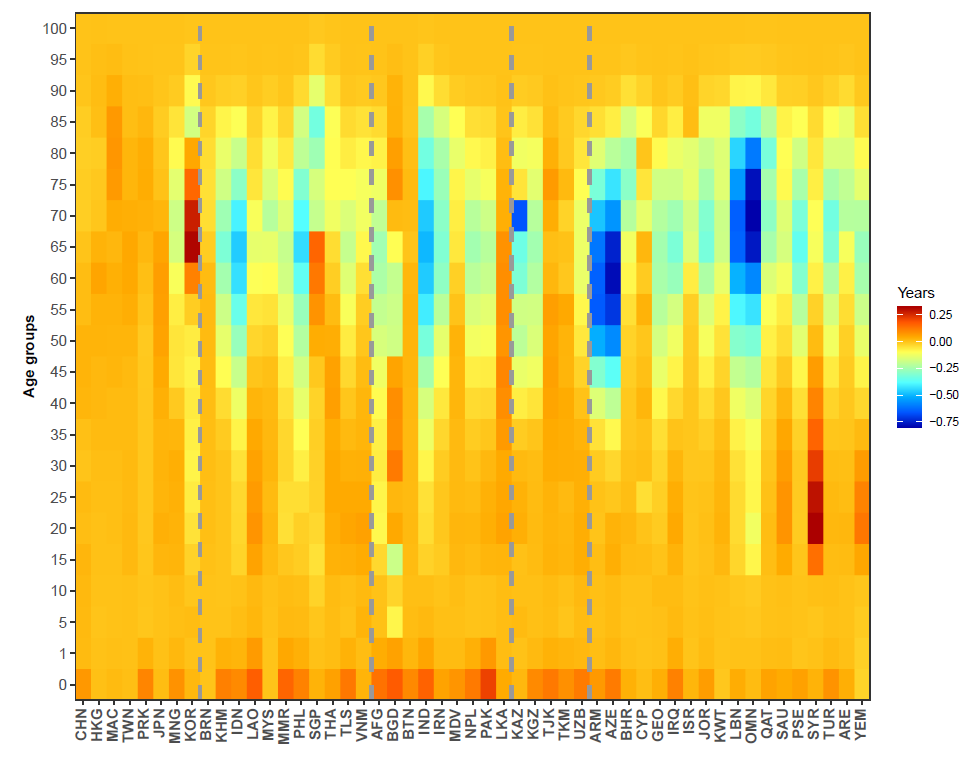


**Note:** The country codes in the figure follow the order below. The names of countries and territories are consistent with those used by the United Nations. **Eastern Asia:** China (CHN), China, Hong Kong SAR (HKG), China, Macau SAR (MAC), China, Taiwan Province of China (TWN), Democratic People's Republic of Korea (PRK), Japan (JPN), Mongolia (MNG), Republic of Korea (KOR); **South-Eastern Asia:** Brunei Darussalam (BRN), Cambodia (KHM), Indonesia (IDN), Lao People's Democratic Republic (LAO), Malaysia (MYS), Myanmar (MMR), Philippines (PHL), Singapore (SGP), Thailand (THA), Timor-Leste (TLS), Viet Nam (VNM); **Southern Asia:** Afghanistan (AFG), Bangladesh (BGD), Bhutan (BTN), India (IND), Iran (Islamic Republic of) (IRN), Maldives (MDV), Nepal (NPL), Pakistan (PAK), Sri Lanka (LKA); **Central Asia:** Kazakhstan (KAZ), Kyrgyzstan (KGZ), Tajikistan (TJK), Turkmenistan (TKM), Uzbekistan (UZB); **Western Asia:**  Armenia (ARM), Azerbaijan (AZE), Bahrain (BHR), Cyprus (CYP), Georgia (GEO), Iraq (IRQ), Israel (ISR), Jordan (JOR), Kuwait (KWT), Lebanon (LBN), Oman (OMN), Qatar (QAT), Saudi Arabia (SAU), State of Palestine (PSE), Syria (SYR), Türkiye (TUR), United Arab Emirates (ARE), Yemen (YEM).
